# Noninvasive Pediatric Liver Fibrosis Measurement: Two-Dimensional Shear Wave Elastography Compared With Transient Elastography

**DOI:** 10.3389/fped.2022.849815

**Published:** 2022-04-28

**Authors:** Léa Chantal Tran, Delphine Ley, Gurvan Bourdon, Stéphanie Coopman, Héloïse Lerisson, Céline Tillaux, Hélène Béhal, Frédéric Gottrand, Madeleine Aumar

**Affiliations:** ^1^University of Lille, Inserm, CHU Lille, U1286 - INFINITE - Institute for Translational Research in Inflammation, Lille, France; ^2^Division of Gastroenterology, Hepatology and Nutrition, Department of Paediatrics, Jeanne de Flandre Children's Hospital, CHU Lille and University of Lille, Lille, France; ^3^Department of Paediatric Imaging, Jeanne de Flandre Children's Hospital, CHU Lille, Lille, France; ^4^University of Lille, CHU Lille, ULR 2694 - METRICS: Évaluation des Technologies de Santé et des Pratiques Médicales, Lille, France

**Keywords:** liver disease, pediatric, shear wave elastography, cirrhosis, ultrasound

## Abstract

**Objectives:**

Although transient elastography (TE) is the primary noninvasive method for assessing liver fibrosis, its use remains to be validated in children. This study aims to evaluate the agreement between two-dimensional ultrasound shear wave elastography (2D-SWE) and TE to assess pediatric liver stiffness method.

**Methods:**

During the 18-month study, we prospectively included 101 consecutive children (median age: 8.5 years, range: 1 month to 17 years) who required TE for medical reasons, and in whom 2D-SWE measurement was performed within a 3-month follow-up during a routine ultrasound. Liver elasticity values were classified according to the Metavir score using published pediatric norms for TE and according to the manufacturer's reference values for 2D-SWE. The Spearman's correlation coefficient was used to assess the relationship between the elasticity measured by the two techniques. Concordance was described by the Bland–Altman method.

**Results:**

A strong correlation (rho = 0.70, *p* < 0.001) was found between 2D-SWE and TE for the elasticity measures. The strength of correlation was higher among patients older than 6 years (rho = 0.79, *p* < 0.001). Concordance between liver fibrosis stages assessed by these techniques was moderate [weighted kappa = 0.46, (95% CI: 0.35–0.57)]. When considering stages over F2, 2D-SWE diagnostic performances showed a sensitivity of 85% (95% CI: 74–92) and a specificity of 57% (95% CI: 42–70) compared with TE.

**Conclusion:**

Measurements of the liver stiffness using 2D-SWE and TE are strongly correlated. The moderate concordance between these techniques for assessing the liver fibrosis stage provides evidence against alternating between these methods during follow-up of patients with the chronic liver diseases.

## Introduction

Chronic liver diseases and surgery affecting the liver, such as the Fontan procedure and liver transplantation, can result in liver fibrosis. The severity of complications associated with the development of cirrhosis and portal hypertension emphasize the need for accurate assessment of liver disease progression, to optimize patient management and improve therapeutic strategies. Though it is the gold-standard technique for evaluating degree of liver fibrosis, liver biopsy for histology is invasive, expensive, nonrepeatable and carries a high risk of complications. In the past decades, noninvasive methods to assess and monitor liver fibrosis in children have been developed (1). Elastography methods have emerged as interesting alternatives to liver biopsy in the pediatric studies conducted on healthy patients or with various stages of liver fibrosis (2–4), particularly in biliary atresia (5, 6), obesity (7, 8) or Wilson's disease (9, 10). Transient elastography (TE) is mechanic impulse elastography that has been validated against pediatric liver biopsy to evaluate histopathologic fibrosis stage in several indications (e.g., hepatitis, biliary atresia, nonalcoholic steatohepatitis) (11–13). TE relies on vibration waves and is classified as 1-dimensional shear-wave elastography without direct image guidance. Two-dimensional shear wave elastography (2D-SWE) is a more recent technique that measures the velocity of elastic shear weaves in the liver tissue during routine liver ultrasound (1). To date, 2D-SWE liver elasticity measure has not been validated in children (14).

The aim of this study was to assess the correlation and concordance between 2D-SWE and TE in the evaluation of liver fibrosis, for assessment of elasticity measures and fibrosis severity, in children with chronic liver disease.

## Methods

### Ethics Approval and Informed Consent

Patients and their parents/caregivers were informed about this study by the medical doctor who prescribed or performed the TE and gave their consent to participate. Data were collected according to the research protocol, good clinical practice, and relevant laws and regulations in France. The study did not require IRB approval due to its observational, noninterventional design. The study was approved by the Commission Nationale de l'Informatique et des Libertés (CNIL-French Data Protection Authority).

### Study Sample

We conducted an 18-month prospective, monocentric, cross-sectional, noninterventional study at the Lille University Jeanne de Flandre Children's Hospital. All the patients younger than 18 years, who required a TE and then had abdominal ultrasound with 2D-SWE assessment within a 3-month follow-up period, were included. They all had a follow-up for a liver pathology, a disease or medical condition at risk of hepatic complication (prolonged total parenteral nutrition, chemotherapy), and abnormal hepatic blood test needing further investigations (cytolysis and cholestasis). Patients were excluded when they did not have ultrasound with measurement of liver elasticity using 2D-SWE within 3 months before or after TE or if they have already been included.

The following characteristics were collected: age, sex, weight, height, pathology, and clinical signs of hepatic fibrosis complications (portal hypertension and hepatic failure). Body mass index (BMI) *z*-score was calculated using AnthroPlus version 1.0.4. software (with WHO standard curves). Serum aspartate aminotransferase and platelets count within 3 months before or after TE measurement were collected, when available. We calculated the aspartate aminotransaminase-to-platelets ratio index (APRI) score, which was then weighted by BMI *z*-score to create a modified APRI (M-APRI) using the formula: M-APRI = APRI × (BMI z-score) (15).

### Liver Stiffness Assessment

Liver stiffness was measured using both the techniques at the last or second-last right intercostal space on the midaxillary line. Patients were placed in the supine position, with their right hand under their head, when possible. Ten measurements (expressed in kPa) were acquired per patient and the mean value was calculated.

### Transient Elastography

Transient elastography (Fibroscan^®^ EchoSens, Paris, France) was performed by a pediatric gastroenterologist using probes adapted to the patient's chest morphology: S1 (chest perimeter < 45 cm), S2 (chest perimeter 45–75 cm), or M (chest perimeter > 75 cm). The results were plotted with the Metavir score according to the reference values validated in children (16).

### Two-Dimensional Ultrasound Shear Wave Elastography

Abdominal ultrasound was performed by a pediatric radiologist with an Applio 500^®^ (Toshiba Medical Systems Corporation, Odawara, Japan) using a PVT 375 BT/SC abdominal probe. The results were interpreted using the manufacturer's reference values and their equivalent Metavir scores. Cutoffs to classify liver fibrosis stages are given in Table 1.

**Table 1:** Liver fibrosis stage according to the liver stiffness measured by transient elastography [according to Fitzpatrick et al. (16)] and two-dimensional shear wave elastography per Toshiba Medical (unpublished data)].

**Metavir score**	**Transient elastography (kPa)**	**2D-SWE** **(kPa)**
**F0**	<6.1	<7
**F1**	6.1–6.9	7–9
**F2**	6.9–7.5	9–11.5
**F3**	7.5–14.1	11.5–14.5
**F4**	> 14.1	> 14.5

*kPa, kilopascal*.

### Statistical Analysis

Continuous and categorical variables are expressed as median [interquartile range (IQR)] and number (percentage), respectively. Normality of distributions was assessed using histograms and the Shapiro–Wilk test.

Spearman correlation coefficients were used to assess the relations between TE- and 2D-SWE-measured elasticity and risk of liver fibrosis.

Concordance was visualized between the elasticity measured by TE and by 2D-SWE using the Bland–Altman method. This approach allows to describe agreement between to quantitative measurements using means and standard deviations values of the differences between these two measurements (17, 18). Level of agreement between liver fibrosis stages determined by the two techniques was evaluated using the weighted kappa (*k*) coefficient and its 95% CI, where *k* < 0 indicated no agreement, 0–0.20 slight agreement, 0.21–0.40 fair agreement, 0.41–0.60 moderate agreement, 0.61–0.80 substantial agreement, and 0.81–1.00 almost perfect agreement.

The predictive value of elasticity measured by 2D-SWE was assessed using the area under the receiver operating characteristics (ROC) curve (AUC) and the optimal threshold was determined by maximizing the Youden index. Threshold sensitivities and specificities are expressed with 95% CIs.

Statistical testing was performed at the two-tailed α level of 0.05. Data were analyzed using the SAS software package, release 9.4 (SAS Institute, Cary, North Carolina, USA).

## Results

### Study Sample

Among 192 eligible patients who had TE measurements during the study period, 91 were excluded: 89 did not have 2D-SWE measurements within 3 months and 2 had undergone two measurements. For the 101 patients (49.5% boys), median age was 8.5 years (range: 1 month−17 years) and most (62%, *n* = 63) were age 6–18 years. Liver disease etiology and patient ages are reported in Table 2. For TE assessment, 7 patients needed an S1 probe, 73 an S2 probe, and 19 an M probe (information missing for 2 patients). In total, twenty-four patients (24%) had clinical signs of portal hypertension, among whom 10 had splenomegaly, 12 had esophageal varices, and 7 had ascites. Two patients (2%) displayed signs of liver failure. Only one patient had a previous liver transplantation for biliary atresia. Only 4 patients in this sample had simultaneous liver biopsy and stiffness measurement, for suspicion of autoimmune hepatitis, which was histologically confirmed.

**Table 2:** Patient age, APRI, TE, and 2D-SWE median values according to Metavir score and liver disease.

**Liver disease**	**Age (years) median [min–max]**	**APRI median [min–max]**	**Transient elastography (kPa) median [min–max]**					**2D-SWE (kPa) median [min–max]**				
			**F0**	**F1**	**F2**	**F3**	**F4**	**F0**	**F1**	**F2**	**F3**	**F4**
Congenital biliary^*^ diseases (*n =* 16)	6	0.7	4.7	6.5	N/A	11.9	59.1	5	7.7	10.3	13	27
	[0.1–18]	[0.2–4.9]	[3.2–4.7]	[6.2–6.5]		[9.5–13.5]	[14.4–75]	[4–6.5]	[7.3–8.8]	[10–11.3]	[12,13]	[16–106]
			*n =* 4	*n =* 2		*n =* 4	*n =* 6	*n =* 2	*n =* 2	*n =* 3	*n =* 2	*n =* 7
TPN-associated liver disease (*n =* 13)	5.3	0.3	3.9	6.3	7.4	8.4	16.4	5.8	7	10.1	12.2	15.8
	[0.3–14.6]	[0.1–0.6]	[3.2–5.9]	[6.1–6.4]		[7.9–8.9]		[5-6]		[9-11]	[12–12.4]	
			*n =* 7	*n =* 2	*n =* 1	*n =* 2	*n =* 1	*n =* 4	*n =* 2	*n =* 4	*n =* 2	*n =* 1
Fontan surgery (*n =* 13)	8.3	0.3	N/A	6.8	N/A	13	19.4	N/A	N/A	11	13	17
	[4.1–16.8]	[0.1–0.6]				[9.3–13]	[14.6–32.4]				[12-14]	[17-20]
				*n =* 1		*n =* 3	*n =* 9			*n =* 3	*n =* 7	*n =* 3
Inherited metabolic diseases (*n =* 9)	5.3	0.3	6.3	7.9	10.4	14	N/A	4.2	6.1	N/A	7.7	14.4
	[1.4–16]	[0.2–0.8]	[5–6.3]	[7–7.9]	[10.2–10.9]			[3.5–5]			[7.7–9]	
			*n =* 3	*n =* 3	*n =* 2	*n =* 1		*n =* 4	*n =* 1		*n =* 3	*n =* 1
Congenital hepatic fibrosis (*n =* 7)	5.3	0.2	4.6	6.6	N/A	N/A	19.5	7	7.9	9.5	13.5	42
	[1.3–16.3]		[3.1–4.9]				[17–21.9]			[9,10]	[13,14]	
			*n =* 4	*n =* 1			*n =* 2	*n =* 1	*n =* 1	*n =* 2	*n =* 2	*n =* 1
Autoimmune hepatitis (*n =* 6)	11.9	1.2	3.6	6.1	N/A	10.7	24.3	5.4	7.8	N/A	12	17.5
	[7.8–13.5]	[0.2–2.2]	[3.1–4.1]				[19.6–28.9]					[17,18]
			*n =* 2	*n =* 1		*n =* 1	*n =* 2	*n =* 1	*n =* 2		*n =* 1	*n =* 2
Cystic fibrosis (*n =* 6)	12.3	0.6	N/A	7.4	9.2	13.3	16	N/A	N/A	7.3	10.4	24.2
	[9.2–13.7]	[0.2–1.9]			[9–10.4]					[7.1–7.4]		[14.5–55.6]
				*n =* 1	*n =* 3	*n =* 1	*n =* 1			*n =* 2	*n =* 1	*n =* 3
Viral hepatitis (*n =* 6)	11.5	0.5	4.9	N/A	N/A	7.6	N/A	5.9	7	9	N/A	N/A
	[3.7–14.7]		[4,5]					[5.4–6.4]	[7–8.7]			
			*n =* 5			*n =* 1		*n =* 2	*n =* 3	*n =* 1		
Sclerosing cholangitis (*n =* 5)	14.2	1.3	4.80	N/A	N/A	N/A	25.2	N/A	N/A	10.3	14.4	70.5
	[9.3–16.9]	[0.2–4.8]	[3.8–5.7]				[17–48]			[9.5–11]		[14.4–48]
			*n =* 2				*n =* 3			*n =* 2	*n =* 1	*n =* 2
Various^**^ (*n =* 20)	6.8	1.2	4.9	6.5	N/A	9.1	31.6	6.3	7.5	9.9	13	16.1
	[0.1–16]	[0.07–8.1]	[3.2–5.5]			[8.2–11.7]	[15–75]	[6–6.8]	[7.1–8.3]	[9.1–11]	[12-14]	[12.9–74]
			*n =* 9	*n =* 1		*n =* 6	*n =* 4	*n =* 4	*n =* 3	*n =* 8	*n =* 2	*n =* 3

*2D-SWE, two-dimensional shear wave elastography; APRI, aspartate aminotransaminase-to-platelet ratio index; n, number; kPa, kilopascal; N/A, not applicable; TE, transient elastography; TPN, total parenteral nutrition*.

* *Biliary atresia (n = 10), choledochal cyst (n = 4), mesenchymal hamartoma of the liver (n = 1), unidentified malformation syndrome (n = 1)*.

** *Unexplained liver fibrosis (n = 4), abetalipoproteinemia (n = 4), unexplained cholestasis (n = 1), Alagille syndrome (n = 2), progressive familial intrahepatic cholestasis (n = 1), unexplained cytolysis (n = 3), obesity-induced hepatic steatosis (n = 1), chemotherapy-associated liver fibrosis (n = 1), Beals syndrome (n = 1), Turner syndrome (n = 1), congenital dyskeratosis (n = 1)*.

### Liver Stiffness Measured by TE and 2D-SWE

Liver stiffness values from the two techniques, according to pathology and Metavir score, are given in Table 2. Most measures were from 5 to 10 kPa per both TE and 2D-SWE.

Transient elastography and 2D-SWE were strongly correlated for the overall sample, and more strongly in those over age 6 years (rho = 0.79, *p* < 0.001), while the association was moderate among patients 6 years or younger (rho = 0.49, *p* =0.002). Similarly, in patients with liver fibrosis stage ≥F2, the correlation between TE and 2D-SWE values was strong. The same strong correlation coefficient was found in patients older than 6 years, but was moderate for patients 6 years or younger (Table 3).

**Table 3:** Correlation between the liver stiffness measurements between transient elastrography and 2D-SWE, and between TE and APRI.

	**Spearman correlation (*p*-value)**
**TE and 2D-SWE**	
** All stages of liver fibrosis**	
All patients	0.70 (*p* < 0.001)
Age < 6 years	0.49 (*p =* 0.002)
Age ≥ 6 years	0.79 (*p* < 0.001)
** According to stages of liver fibrosis using Metavir scoring**	
All patients	0.69 (*p* < 0.001)
Age < 6 years	0.56 (*p =* 0.019)
Age ≥ 6 years	0.69 (*p* < 0.001)
**TE and APRI**	0.43 (*p =* 0.001)
**TE and APRI using BMI** ***z*****-score**	0.34 (*p =* 0.019)

*2D-SWE, two-dimensional ultrasound shear wave elastography; 2-APRI, aspartate aminotransaminase-to-platelets ratio index*.

**Figure 1:**
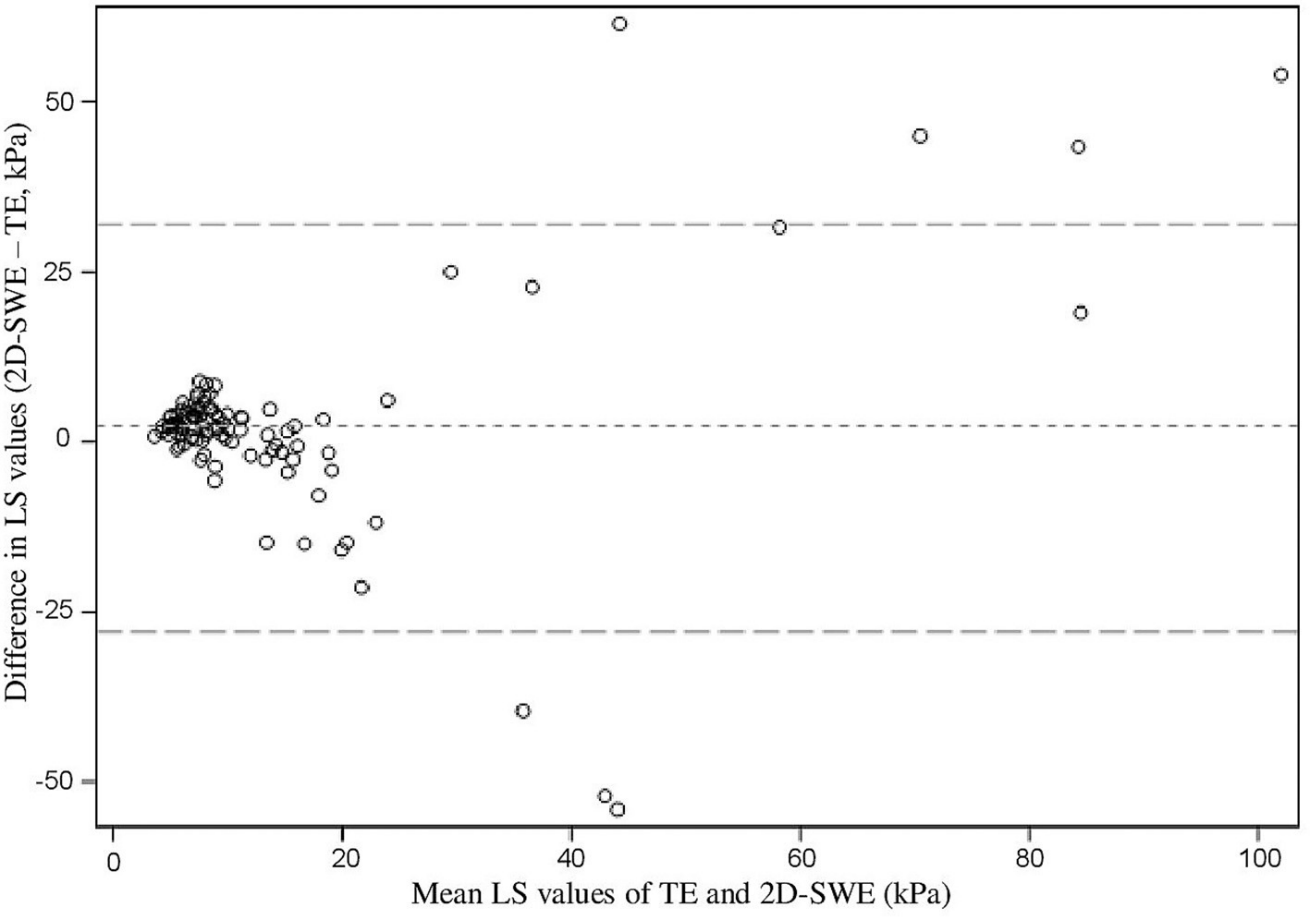
Bland-Altman plot showing differences in liver stiffness values according to the mean of hepatic elasticity evaluated by TE and 2D-SWE, two-dimensional shear wave elastography; kPa, kilopascal; LS, liver stiffness; TE, transient elastography.

Because of the relatively small subgroup sizes, analyzing patients according to pathology/group of pathologies or BMI was not possible. As shown in Figure 1, the higher the elasticity vales, the greater the gap between 2D-SWE and TE values. When considering fibrosis stage, concordance between TE and 2D-SWE values was moderate (weighted kappa = 0.46; 95% CI: 0.35–0.57).

Sensitivity analysis after excluding the 13 patients with Fontan-associated liver disease (FALD) showed similar results [correlation coefficient of 0.64 (*p* < 0.001) between TE and 2D-SWE].

### Diagnostic Performances of TE and 2D-SWE for Predicting Liver Fibrosis Stage

Using the 2D-SWE manufacturer's diagnostic thresholds compared with TE, sensitivity and specificity of 2D-SWE values to determine stages of liver fibrosis ≥ F2 were 85% (95% CI: 74–92) and 57% (95% CI: 42–70), respectively. Detailed AUC, sensitivity, and specificity values are given in Table 4.

**Table 4:** Diagnostic performances of 2D-SWE compared with TE as reference method, according to Metavir score.

**Metavir score**	**Area under the curve**	**Liver stiffness threshold value (kPa) using 2D-SWE**	**Sensitivity (%)**	**Specificity (%)**
**≥F1**	0.78	7.1	89	41
**≥F2**	0.85	9.8	82	74
**≥F3**	0.86	9.8	84	73
**≥F4**	0.92	13	84	91

*The 2D-SWE liver stiffness threshold value is the optimal based on the highest Youden index. TE, transient elastography; 2D-SWE, two-dimensional shear wave elastography; kPa, kilopascal*.

### Correlations Between TE and APRI

The correlation coefficient rho between risk of liver fibrosis estimated by the APRI test and TE was weak, as well as for the modified APRI using BMI z-score (Table 3).

## Discussion

To the best of our knowledge, this is the first study comparing 2D-SWE and TE in a large pediatric sample across a wide age range. Liver elasticity measured by 2D-SWE and TE were strongly correlated, especially in patients older than 6 years. Concordance between the two techniques for defining liver fibrosis stage was moderate.

Although it is the gold-standard liver fibrosis evaluation, liver biopsy is an invasive procedure associated with risk of complications such as bleeding, bowel perforation, and fistula. Recently, new noninvasive alternatives for evaluating the liver fibrosis stage have been developed. These combine clinical scores, blood tests, and imaging: B-AST (BMI *z*-score × aspartate transaminase) (15), APRI (19), acoustic radiation force impulse elastography (ARFI) (20, 21), point-SWE, and magnetic resonance elastography (14). However, validations of these markers in pediatric patients are needed. TE is the only validated method in children with established reference values to stage liver fibrosis (16). 2D-SWE provides qualitative and quantitative evaluation of liver elasticity and morphological, real-time 2D imaging. Unlike the TE probe, 2D-SWE does not compress the hepatic tissue, avoiding variations in stiffness values (22). 2D-SWE also has a wider bandwidth, allowing greater distinction of fibrosis stage (23).

Strong, significant correlations between TE- and 2D-SWE-based fibrosis stage measures have been reported in adult cohorts [i.e., rho = 0.83 (24), rho = 0.69 (25), and rho = 0.93 (26)]. In children, liver fibrosis assessment in chronic liver diseases using 2D-SWE compared with liver histology showed good-to-moderate correlations with fibrosis stage [i.e., rho = 0.83 (3) and rho = 0.43 (27)]. Although many authors have described use of 2D-SWE and TE in specific pediatric liver diseases such as biliary atresia (5, 28), obesity (7), and Wilson disease (9), only one previous study reported a comparison of these two methods in various chronic liver diseases (21) (see Supplementary Table). The moderate correlation herein between 2D-SWE and TE in children 6 years or younger could be a result of having a larger number of patients with no or low liver fibrosis in this age group and/or the narrower range of value for stages < F2 compared with those > F3. The impact of age on the liver elasticity measurement has been demonstrated in healthy children using TE (29). The lower correlation herein might also be explained by the fact that it is more difficult to perform these techniques in younger children (e.g., poorer cooperation, smaller thoracic morphology and liver, narrower intercostal space), which may make use of the probe more challenging and reduce measurement reliability (30).

Confounding factors influencing diagnostic accuracy and reproducibility of liver elastography measurements in children, including constitutive factors (age, gender, and BMI), technical factors (probe and right vs. left liver lobe) and disease etiology have been well described (1, 30). However, defining a reference measurement location is impossible because of the particularities associated with some liver pathologies (e.g., right lobe assessment is impossible for left liver transplant and right lobe measurements are more accurate in patients with cystic fibrosis).

Strong agreement between 2D-SWE and TE in the evaluation of liver fibrosis has been previously studied in a few adult cohorts (31–33) and two pediatric studies (9, 21). Herein, we found a moderate concordance for liver fibrosis stages between 2D-SWE and TE, which could be explained by the technical differences between these methods (34) that may hinder direct comparison. 2D-SWE mechanically assesses horizontal wave propagation, whereas TE is an ultrasonic procedure that evaluates vertical wave propagation. Unlike 2D-SWE, which allows exploration of a large hepatic surface, TE does not display the aspect of the wave emission area, disturbing the analysis of heterogenous liver fibrosis. TE is highly dependent on the operator, whereas 2D-SWE can display internal validation, allowing greater inter- and intrarater reliabilities (30, 35).

Sensitivity and specificity of 2D-SWE compared with TE were 85 and 57%, respectively, for liver fibrosis stages ≥ F2. Compared with liver biopsy, Cassinotto et al. showed higher diagnostic performance of 2D-SWE (sensitivity 83%, specificity 82%, positive predictive value 88%, and negative predictive value 75%) in adult patients with liver fibrosis ≥ F2 (31). In the study by Belei, sensitivity of 2D-SWE compared with TE was 92.9% for F1, 83.3% for F2, 87.5% for F3, and 85.7% for F4 (21). However, these values are highly dependent on both the defined fibrosis stage thresholds and the criteria for selecting sensitivity values (threshold maximizing either sensitivity or specificity, both or that with the greatest clinical relevance). In a meta-analysis of 12 studies with a cumulative 550 pediatric patients with liver fibrosis, 2D-SWE demonstrated overall sensitivity and specificity of 96 and 87%, respectively, compared with several fibrosis staging systems (Metavir, Ishak, Batts-Ludwig, and Brunt) converted into a single unified staging system, which were significantly higher than ARFI (28). These cumulative data support the superior diagnostic performance of 2D-SWE compared with other elastography methods.

The major strengths of this study include the large sample size from a single center at which the same measurement techniques were used with the equipment by trained physicians. 2D-SWE was performed by only two operators, avoiding an operator effect. The relatively brief interval between TE and 2D-SWE avoided variations because of evolution of the underlying pathology. Nevertheless, the study was not without limitations. The heterogeneous range of pathologies disallowed analyses of subgroups, children younger than 2 years and those with extreme weight values, who were insufficiently represented. Pediatric reference values for liver stiffness using 2D-SWE compared with liver biopsy have been recently proposed only in healthy children, without clear consensus (23, 29, 36). Using the manufacturer's (Toshiba Medical) reference values, which were derived from a preliminary study in adults with hepatitis C, presents a major bias. Another potential bias is our cohort's subgroup of patients with FALD. Hepatic congestion increases TE values, which could be falsely attributed to fibrosis (37, 38). To address this bias, we performed a sensitivity analysis and confirmed the absence of significant differences after excluding 13 children with FALD. Our cohort included only one patient with obesity-induced hepatic steatosis, making it nonrepresentative of the overall pediatric population with chronic liver diseases.

## Conclusion

Further validations of 2D-SWE must be confirmed before its use in the clinical practice, with particular focus on follow-up of liver fibrosis for patients older than 6 years. Reference values for chronic liver diseases using 2D-SWE need to be established. Based on the current evidence, children aged 6 years or younger at diagnosis are better assessed by TE and the same technique should be used at each follow-up.

## Data Availability Statement

The raw data supporting the conclusions of this article will be made available by the authors, without undue reservation.

## Ethics Statement

Ethical review and approval was not required for the study on human participants in accordance with the local legislation and institutional requirements. Written informed consent to participate in this study was provided by the participants' legal guardian/next of kin.

## Author Contributions

SC, GB, FG, and MA conceived and designed the study. LT, SC, GB, HL, CT, HB, and MA did the analysis and interpretation of the data. LT, SC, GB, HB, FG, and MA drafted of the article. SC, GB, DL, HB, FG, and MA brought critical revision of the article for important intellectual content. All authors approved the final version of the article.

## Conflict of Interest

The authors declare that the research was conducted in the absence of any commercial or financial relationships that could be construed as a potential conflict of interest.

## Publisher's Note

All claims expressed in this article are solely those of the authors and do not necessarily represent those of their affiliated organizations, or those of the publisher, the editors and the reviewers. Any product that may be evaluated in this article, or claim that may be made by its manufacturer, is not guaranteed or endorsed by the publisher.
