# Supplementary material for: Noninvasive Pediatric Liver Fibrosis Measurement: Two-Dimensional Shear Wave Elastography Compared With Transient Elastography
Source: Front Pediatr. 2022 Apr 28;10:849815. doi: 10.3389/fped.2022.849815 (PMC9095976; doi:10.3389/fped.2022.849815)
Supplement: Supplementary file 1 [file Table_1.DOCX]

**Supplemental Table.** Comparison of previous TE and 2D-SWE reports. 2D-SWE: two-dimensional shear wave elastography; BMI: body mass index; N/A: not applicable; TE: transient elastography

| **Study** | **Liver diseases** | **Sample size** | **Mean age (years)** | **Mean BMI (kg/m²)** | **2D-SWE compared with TE results** |
| --- | --- | --- | --- | --- | --- |
| Hwang et al., 2021 (21) | Biliary atresia after Kasai | 63 | N/A | N/A | rho=0.79, p<0.001 |
|  |  |  |  |  |  |
| Marginean et al., 2019 (22) | Obesity | 77 | 10 | 27 | Significantly higher liver stiffness and velocity values |
|  |  |  |  |  |  |
| Hwang et al., 2020 (23) | Wilson disease | 45 | 16 | 20 | rho=0.75, p<0.001 |
|  |  |  |  |  |  |
| Belei et al., 2016 (12) | Various chronic liver diseases | 54 | N/A | N/A | kappa=0.84, p=0.001 |
|  |  |  |  |  |  |
| *Present study* | Various chronic liver diseases | 101 | 9 | 17 | rho=0.70, p<0.001 |
